# A Novel Mechanism of Soluble HLA-G Mediated Immune Modulation: Downregulation of T Cell Chemokine Receptor Expression and Impairment of Chemotaxis

**DOI:** 10.1371/journal.pone.0011763

**Published:** 2010-07-23

**Authors:** Fabio Morandi, Elisa Ferretti, Paola Bocca, Ignazia Prigione, Lizzia Raffaghello, Vito Pistoia

**Affiliations:** Laboratory of Oncology, G. Gaslini Children's Hospital, Genoa, Italy; University of Toronto, Canada

## Abstract

**Background:**

In recent years, many immunoregulatory functions have been ascribed to soluble HLA-G (sHLA-G). Since chemotaxis is crucial for an efficient immune response, we have investigated for the first time the effects of sHLA-G on chemokine receptor expression and function in different human T cell populations.

**Methodology/Principal Findings:**

T cell populations isolated from peripheral blood were stimulated in the presence or absence of sHLA-G. Chemokine receptors expression was evaluated by flow cytometry. sHLA-G downregulated expression of i) CCR2, CXCR3 and CXCR5 in CD4^+^ T cells, ii) CXCR3 in CD8^+^ T cells, iii) CXCR3 in Th1 clones iv) CXCR3 in TCR Vδ2γ9 T cells, and upregulated CXCR4 expression in TCR Vδ2γ9 T cells. sHLA-G inhibited *in vitro* chemotaxis of i) CD4^+^ T cells towards CCL2, CCL8, CXCL10 and CXCL11, ii) CD8^+^ T cells towards CXCL10 and CXCL11, iii) Th1 clones towards CXCL10, and iv) TCR Vδ2γ9 T cells towards CXCL10 and CXCL11. Downregulation of CXCR3 expression on CD4+ T cells by sHLA-G was partially reverted by adding a blocking antibody against ILT2/CD85j, a receptor for sHLA-G, suggesting that sHLA-G downregulated chemokine receptor expression mainly through the interaction with ILT2/CD85j. Follicular helper T cells (T_FH_) were isolated from human tonsils and stimulated as described above. sHLA-G impaired CXCR5 expression in T_FH_ and chemotaxis of the latter cells towards CXCL13. Moreover, sHLA-G expression was detected in tonsils by immunohistochemistry, suggesting a role of sHLA-G in local control of T_FH_ cell chemotaxis. Intracellular pathways were investigated by Western Blot analysis on total extracts from CD4+ T cells. Phosphorylation of Stat5, p70 s6k, β-arrestin and SHP2 was modulated by sHLA-G treatment.

**Conclusions/Significance:**

Our data demonstrated that sHLA-G impairs expression and functionality of different chemokine receptors in T cells. These findings delineate a novel mechanism whereby sHLA-G modulates T cell recruitment in physiological and pathological conditions.

## Introduction

The classical HLA molecules, also known as HLA-class Ia molecules, are extremely polymorphic molecules belonging to the immunoglobulin superfamily [1]. HLA-class Ia molecules are widely but not ubiquitously expressed. Each HLA class Ia molecule consists of a single heterotrimer of heavy chain, β2-microglobulin and a peptide epitope of eight to ten amino acids embed in the peptide-binding groove of the heavy chain. Most peptide epitopes are derived from proteins that are synthesized in the cell, digested by antigen-processing machinery and loaded into the peptide-binding groove. These peptides are presented to antigen-specific T cells through the interaction with T-cell receptor, leading to the killing of cells that are infected with viruses or intracellular bacteria, or tumor cells[1].

Like HLA class Ia molecules, “non-classical” HLA class Ib molecules HLA-E, -F, -G and H can associate with β2-microglobulin (β2m) and can present peptides. However, in contrast with HLA-Ia molecules, class Ib molecules are oligomorphic, with only few alleles. So far, limited information are available about the function of HLA-F and HLA-H. HLA-E is able to present peptides derived from the leader sequence of HLA-class Ia molecules and HLA-G, giving a “self signal” in cells that express HLA-class I molecules, and may have a role in autoimmunity[2]. HLA-G is expressed not only as a cell surface bound molecule, but also as a soluble moiety in body fluids[3], [4], [5]. Seven different isoforms encoded by alternative splicing of the same mRNA, that include membrane-bound HLA-G1, HLA-G2, HLA-G3, and HLA-G4 and soluble secreted HLA-G5, HLA-G6, and HLA-G7. The major isoforms of HLA-G present in serum are soluble HLA-G1 and HLA-G5 generated either by shedding or proteolytic cleavage of the membrane bound isoform or by secretion of a soluble isoform[6].

In normal tissues, HLA-G shows a limited distribution, being detected only on cytotrophoblast cells (20), thymic epithelial cells (21), cytokine-activated monocytes (22), mature myeloid and plasmacytoid dendritic cells (23), and inflamed muscle fibers (24).

The physiological role of this molecule is to establish immune tolerance at the maternal-fetal interface, abrogating the activity of maternal NK cells against fetal tissue[7]. HLA-G can also present peptides, but it is still unclear whether these peptides are important for host defense against pathogens or they act to stabilize the surface expression of HLA-G[2].

Many immunoregulatory functions have been described in the last years for HLA-G molecules, in particular on T cells, B cells, NK cells and antigen presenting cells. HLA-G molecules induce apoptosis[4], inhibit cell proliferation[8], cytotoxicity[5] and differentiation[9], and modulate cytokine release[10].

HLA-G binds four receptors, i.e. ILT2 (immunoglobulin (Ig)-like transcript 2)/CD85j, ILT4 (Ig-like transcript 4)/CD85d, KIR2D4L (killer inhibitory receptor)/CD158d. ILT2 is broadly expressed by lymphoid and myeloid cells (T and B cells, NK cells, dendritic cells, monocytes, macrophages), whereas ILT4 is myeloid-specific (monocytes, macrophages, dendritic cells), and KIR2DL4 is expressed only on NK cells. ILT2 and ILT4 interact with HLA-G, but also with classical HLA class I molecules (with lower affinity), whereas KIR2DL4 can interact only with HLA-G [11]. All these receptors are expressed at low levels by resting cells and are up-regulated on activated cells or in pathological condition, i.e. viral infection[12].

Moreover, LeMaoult et al. have demonstrated that HLA-G can up-regulate the expression of its own receptors, since these receptors were found to be overexpressed in the same pathologies in which HLA-G is upregulated (i.e. AIDS, tumors, autoimmune diseases)[13].

CD160 a glycosylphosphatidylinositol-anchored member of the immunoglobulin superfamily, have been recently described as a receptor for HLA-G. Fons et al. have demonstrated that interaction between soluble HLA-G and CD160 on endothelial cells lead to apoptosis of the latter cells and inhibition of angiogenesis[14]. However, CD160 is expressed not only on endothelial cells, but also on NK cells, NKT cells, T cells [14].

Chemotaxis of immune effector cells is a crucial event for the re-circulation of these cells between lymphoid organs and inflamed tissues[15]. Chemotaxis is mediated by the interaction of chemotactic cytokines (chemokines) with their receptors. Chemokines are highly basic proteins of 70–125 amino acids with molecular masses ranging from 6 to 14 kDa and 20–95% sequence identity each other, with 4 highly conserved cysteine residues. Four chemokines subfamilies have been defined according to the number of amino acids between the first two cysteine residues: CC chemokines, CXC chemokines, XC chemokines and CX3C chemokines. Chemokine receptors are heptahelical G protein-coupled receptors, with a single polypetide chain of 340–380 aminoacids spanning 7 times the surface membrane and with 25–80% sequence identity each other. All receptors display an extracellular acidic N-terminal domain, a serine/threonine-rich intracellular C-terminal domain, and two disulfide bonds in-between the N-terminal domain and the second extracellular loop[16].

Intracellular signalling through G-proteins leads to extension of lamellipodia through cytoskeletal restructuring, shape changes and firm adhesion (chemotaxis), release of oxygen radicals, histamine and cytotoxic granules from granulocytes, and finally modulation of gene transcription. A given chemokine receptor can be inactivated at least through two different mechanisms: i) strong or prolonged chemokine stimulation, leading to uncoupling of G-proteins, binding of arrestin, and internalization of the receptor, or ii) stimulation with an unrelated chemoattractant resulting in the activation of PKA and PKC, phosporylation of the receptor and its inactivation without internalization[17]. Chemokine receptor expression can be modulated by pro-inflammatory (i.e. LPS, TNF, IL-1, IFN-γ) and anti-inflammatory (glucocorticoid hormones, IL-10) *stimuli*, and by cytokines such as IL-4 and IL-13, leading to a modulation of immune cells recruitment at sites of infection and inflammation[18].

So far, no information is available on possible effects of sHLA-G molecules (and other HLA-I molecules) on chemotaxis of immune effector cells.

We have investigated this issue on different T cell populations, not only on classical CD4^+^ and CD8^+^ T cells, but also on TCRγδ^+^ T cells and on follicular helper T cells (T_FH_).

TCRγδ T cells circulate in peripheral blood (3–5% of PBMNC) and express a peculiar TCR composed of γ and δ chains, that recognize phosphoantigens. Most circulating TCRγδ T cells show the Vδ2γ9 rearrangement[19]. T_FH_ are a small subset of TCR αβ T cells present in peripheral blood[20] and secondary lymphoid organs[21]. These cells, that express CXCR5 and ICOS, gain access to the germinal center of secondary lymphoid follicles where they exert a potent helper function for B cell differentiation to antibody secreting cells through IL-21 production[22].

Our data describe a novel immunoregulatory property of sHLA-G molecules, based on the inhibition of chemokine-driven migration of different T cell populations together with downregulation of the expression and function of selected chemokine receptors on these cells.

## Results

### Soluble HLA-G impairs the expression of different chemokine receptors in different T cell populations

We first evaluated by flow cytometry the expression of a panel of CC- and CXC-chemokine receptors in the two major subsets of circulating T lymphocytes, CD4^+^ and CD8^+^ T cells. These receptors were selected on the ground of their physiological relevance[23]. Cells were polyclonally stimulated with anti-CD3 mAb, anti-CD3/CD28 coated beads or PHA, in the presence or absence of sHLA-G. All these *stimuli* produced superimposable results. Therefore, from now onwards, all the experiments described will be those performed using anti-CD3 mAb. The effect of sHLA-G on chemokine receptor expression was lower on resting T cells (not shown).

**Figure 1:**
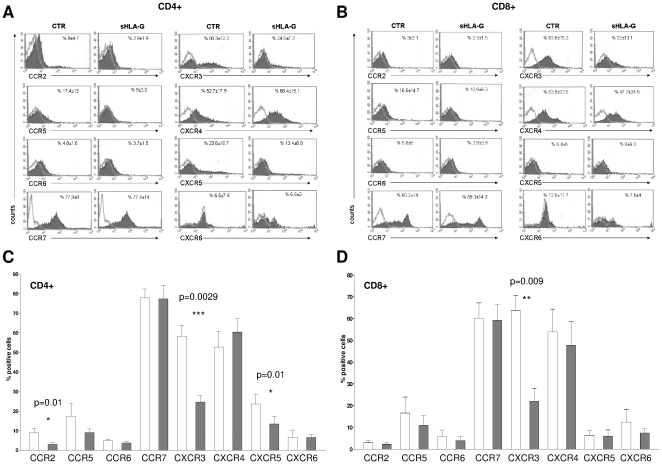
Modulation of chemokine receptors expression in different T cell populations by sHLA-G. Representative histograms of FACS analysis of chemokine receptor expression on CD4^+^ T cells (**panel A**) and CD8^+^ T cells (**panel B**) stimulated with anti-CD3 monoclonal antibody in the presence or absence of sHLA-G (100 ng/ml). Dark profile indicated staining with specific mAb, whereas open profile indicated staining with isotype-matched mAb. Mean and standard deviation of five different experiments is indicated. Histograms show mean of % of positive cells and standard deviation of five experiments performed on CD4+ T cells (**panel C**) and CD8+ T cells (**panel D**). Grey bars indicate cells stimulated in the presence of sHLA-G, white bars indicated cells stimulated with medium alone. Statistical analysis was performed using t test. P values are indicated where the difference is significant.

As shown in Figure 1, panel A, sHLA-G treatment significantly downregulated the expression of CXCR5 (mean %±SD: 23,62±10,7 vs 13,46±8,8, p = 0.01), CCR2 (mean %±SD: 9±4,7 vs 2,95±1,9, p = 0.01) and CXCR3 (mean %±SD: 58,31±12,3 vs 24,68±7,3, p = 0.0029) in CD4+ T cells. In CD8+ T cells, sHLA-G treatment significantly downregulated CXCR3 expression (mean %±SD: 63,82±15,3 vs 22,08±13,1, p = 0.0092) (Fig. 1, panel B). Mean results from five different experiments ±SD are shown in Figs. 1C and 1D).

Next, we investigated the effects of sHLA-G on three polarized subsets of CD4+ T cells, i.e. Th1, Th2 and Th17 cells. These subsets were identified by intracellular staining and flow cytometric analysis according to cytokine production profiles (not shown). In particular, Th1 and Th2 cell clones were >95% IFN-γ^+^/IL-4^−^ and >95% IFN-γ^−^/IL-4^+^, respectively. Bulk Th17 cells enriched from PBMNC were >50% IL17^+^. Chemokine receptor expression in the latter cells was analyzed by flow cytometry gating on cells expressing IL-17A. All these T cell subsets were stimulated in the presence or absence of sHLA-G, and the expression of distinctive chemokine receptors was assessed by flow cytometry[24].

**Figure 2:**
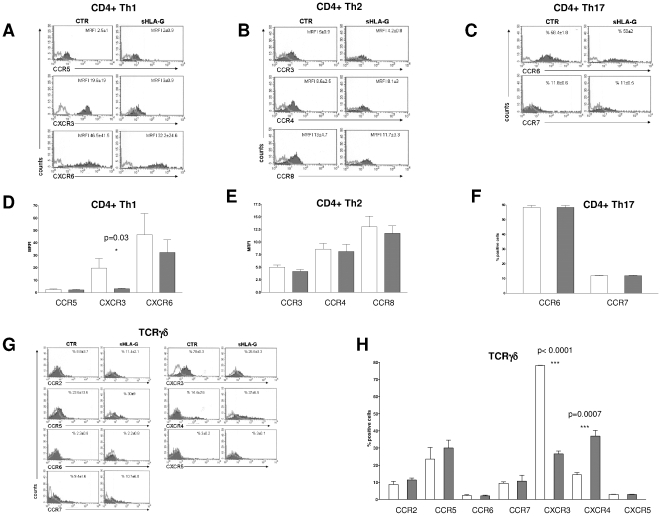
Modulation of chemokine receptors expression in T helper cells and TCR Vδ2γ9 T cells by sHLA-G. Representative histograms of FACS analysis of chemokine receptor expression on CD4^+^ Th1 clones (**panel A**), CD4^+^ Th2 clones (**panel B**), Th17 cells (**panel C**) and TCRγδ T cells (**panel G**) stimulated with anti-CD3 monoclonal antibody in the presence or absence of sHLA-G (100 ng/ml). Dark profile indicated staining with specific mAb, whereas open profile indicated staining with isotype-matched mAb. Mean and standard deviation of five different experiments is indicated. Histograms show mean and standard deviation of five experiments performed on CD4^+^ Th1 clones (**panel D**), CD4^+^ Th2 clones (**panel E**), Th17 cells (**panel F**) and TCRγδ T cells (**panel H**). Grey bars indicate cells stimulated in the presence of sHLA-G, white bars indicated cells stimulated with medium alone. MRFI values are indicated in Panel D and E. % of positive cells is indicated in Panel F and H. Statistical analysis was performed using t test P values are indicated where the difference is significant.

Among Th1-associated chemokine receptors, sHLA-G significantly downregulated the expression of CXCR3 (mean MRFI±SD: 19,58±19 vs 3,03±0,9 p = 0.03) in Th1 clones, whereas CCR5 and CXCR6 were not affected (Fig. 2, panel A). The expression of three Th2-associated chemokine receptors, CCR3, CCR4 and CCR8 was not affected by sHLA-G in Th2 clones (Fig. 2, panel B). In Th17 T cells, sHLA-G treatment did not modulate expression of CCR6 or CCR7, two Th17 associated chemokine receptors (Fig. 2, panel C). Mean results from five different experiments ±SD are shown in Figs. 2D, E and F.

We next investigated modulation of a wide panel of CC- and CXC- chemokine receptors in TCR Vδ2γ9 T cells by sHLA-G (Fig. 2, panel G). A significant up-regulation of CXCR4 (mean %±SD: 14,41±2,6 vs 37±6,5 p = 0.007) was detected, whereas CXCR3 was significantly downregulated (mean %±SD: 78,09±0,3 vs 26,6±3,3, p<0.0001). Mean results from five different experiments ±SD are shown in Fig. 2, panel H).

### Chemotaxis of different T cell populations is dampened by soluble HLA-G treatment

The above T cell populations but Th2 and Th17 cells were next subjected to *in vitro* migration assays using ligands of the chemokine receptors modulated by sHLA-G treatment.

**Figure 3:**
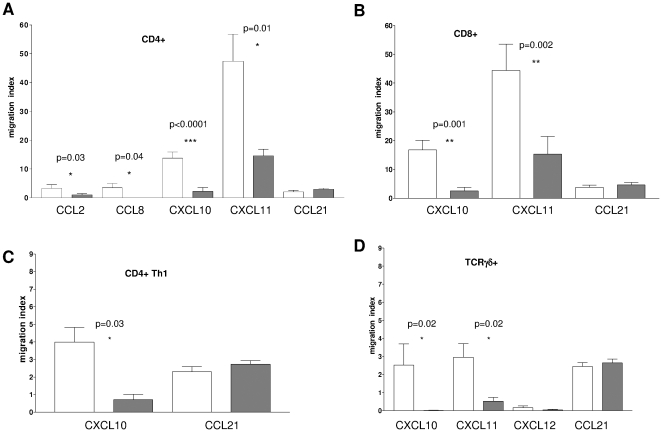
Modulation of chemotaxis of different T cell populations by sHLA-G. CD4+ T cells (**panel A**), CD8+ T cells (**panel B**), Th1 cell clones (**panel C**) and TCR Vδ2γ9 T cells (**panel D**) were stimulated with anti-CD3 monoclonal antibody in the presence (grey bars) or absence (white bars) of sHLA-G (100 ng/ml) and then subjected to i*n vitro* chemotaxis assay using Transwell system. Chemokines were tested at 300 ng/ml. Results are expressed as migration index (number of migrated cells/number of dispensed cells*100). Mean of five different experiments ± SD is shown. Statistical analysis was performed using t test. P values are indicated where the difference is significant.

As shown in Fig. 3, panel A, sHLA-G treatment significantly dampened the chemotaxis of CD4^+^ T cells towards i) two ligands of CCR2, i.e. CCL2 (migration index 2,9 vs 0,7, p = 0.033) and CCL8 (migration index 2 vs 0, p = 0.048) and ii) two ligands of CXCR3, i.e. CXCL10 (migration index 13,77 vs 2,21, p<0,0001) and CXCL11 (migration index 47,40 vs 14,55, p = 0,012). No chemotaxis of CD4^+^ T cells was observed towards CXCL13, the unique ligand of CXCR5, irrespective of the cells had been treated or not with sHLA-G (data not shown).

CD8+ T cell chemotaxis towards CXCL10 and CXCL11 was dampened by sHLA-G treatment, as shown in Fig. 3, panel B (migration index 16,77 vs 2,54, p = 0,001 and 44,37 vs 15,33, p = 0,002, respectively).

Fig. 3, panel C shows that chemotaxis of Th1 T cell clones towards CXCL10 was significantly reduced by sHLA-G treatment (migration index 3,73 vs 0,93, p = 0,03).

As shown in Fig. 3, panel D, TCR Vδ2γ9 T cell chemotaxis towards CXCL10 and CXCL11 was significantly downregulated by sHLA-G (migration index 2,53 vs 0,02, p = 0,02 and 2,96 vs 0,52, p = 0,0024, respectively). Conversely, chemotaxis towards CXCL12, the ligand of CXCR4, was not affected by sHLA-G treatment.

Chemotaxis of all above T cell populations towards CCL21, a ligand of CCR7 (which was not modulated by sHLA-G) was not inhibited by sHLA-G (Figure 3 panel A,B,C and D).

### sHLA-G is expressed in secondary lymphoid follicles and impairs *in vitro* chemotaxis of T_FH_ cells through downregulation of CXCR5 expression

All T cell populations here investigated recirculate from peripheral blood to secondary lymphoid organs. sHLA-G is secreted by different cell types, such as monocytes, dendritic cells and endothelial cells and is detected in sera from normal donors[25].

T_FH_ cells display an unique homing pattern to secondary lymphoid organs where they are attracted to the germinal centres of secondary lymphoid follicles by a gradient of CXCL13[26].

**Figure 4:**
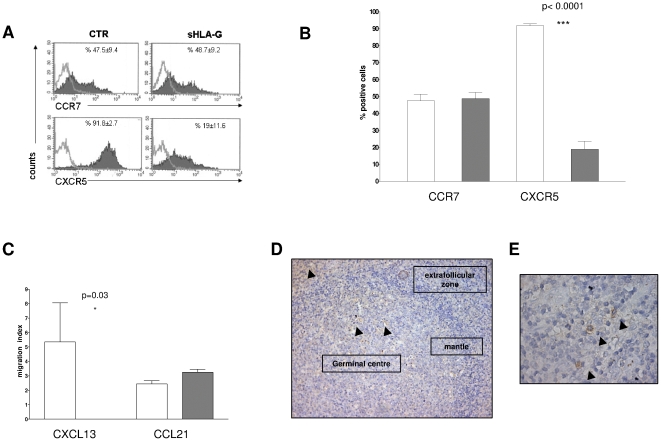
Effects of sHLA-G on follicular helper T cells (T_FH_) from human tonsil. **Panel A.** Representative histograms of FACS analysis of chemokine receptor expression on T_FH_ stimulated with anti-CD3 monoclonal antibody in the presence or absence of sHLA-G (100 ng/ml). Dark profile indicated staining with specific mAb, whereas open profile indicated staining with isotype-matched mAb. Mean and standard deviation of five different experiments is indicated. **Panel B.** Histograms show mean of % positive cells and standard deviation of five experiments performed on T_FH_. Grey bars indicate cells stimulated in the presence of sHLA-G, white bars indicated cells stimulated with medium alone. Statistical analysis was performed using t test. P values are indicated where the difference is significant. **Panel C.** T_FH_ were stimulated with anti-CD3 monoclonal antibody in the presence (grey bars) or absence (white bars) of sHLA-G (100 ng/ml) and then subjected to i*n vitro* chemotaxis assay using Transwell system. Chemokines were tested at 300 ng/ml. Results are expressed as migration index (number of migrated cells/number of dispensed cells*100). Mean of five different experiments ± SD is shown. Statistical analysis was performed using t test. P values are indicated where the difference is significant. **Panel D.** Tonsil tissue sections were stained with an anti-HLA-G1/G5 mAb as detailed in Materials and Methods. Continuous arrows indicated sHLA-G^+^ cells in germinal centre. Dotted arrows indicate sHLA-G^+^ cells in sub-epithelial areas. A magnification is shown in **Panel E**. Arrows indicated sHLA-G^+^ cells.

We evaluated by flow cytometry the expression of two distinctive chemokine receptors, i.e. CXCR5 and CCR7, in T_FH_ cells stimulated in presence or absence of sHLA-G. As shown in Fig. 4, panel A, CXCR5 but not CCR7 expression was significantly dampened by sHLA-G treatment (mean %±SD: 91,8±2,7 vs 19±11,6, p = 0.0013). Mean results from five different experiments ±SD are shown in Fig. 4, panel B. Chemotaxis of T_FH_ cells towards CXCL13 was significantly dampened by sHLA-G treatment (migration index 5,35 vs 0, p = 0.03), whereas migration towards CCL21, a ligand of CCR7, was not (Figure 4, panel C).

To investigate the physiological relevance of our findings, we next evaluated the expression of HLA-G in tonsil tissue sections by immunohistochemical staining with an anti HLA-G1/G5 mAb. As shown in Fig. 4, panel D and E, numerous HLA-G1/G5^+^ cells were detected in germinal centres and sub-epithelial areas (arrows), whereas such cells were virtually absent from the follicular mantle of secondary lymphoid follicles. These findings suggest that trafficking of T_FH_ cells in germinal centres may be physiologically modulated by sHLA-G.

### The inhibitory receptor ILT2/CD85j is involved in sHLA-G mediated inhibition of T cell chemotaxis

**Figure 5:**
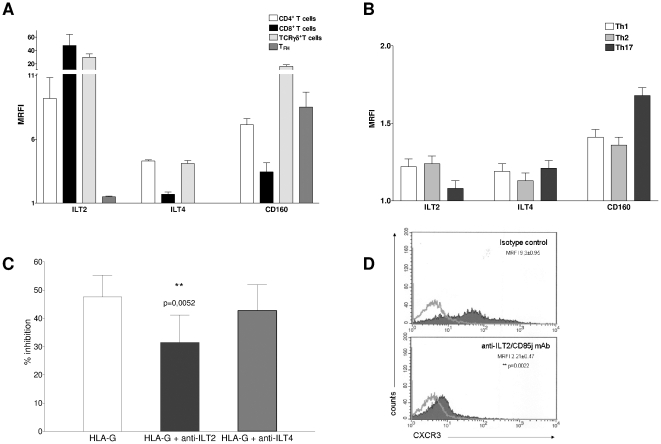
Expression and function of sHLA-G receptors in different T cell populations. **Panel A**. FACS analysis of the expression of sHLA-G receptors ILT2/CD85j, ILT4/CD85b and CD160 in CD4+ T cells (white bars), CD8+ T cells (black bars), TCR Vδ2γ9 T cells (light grey bars) and T_FH_ cells (grey bars). Results are expressed as MRFI. Mean of five different experiments ± SD is shown. **Panel B**. FACS analysis of the expression of sHLA-G receptors ILT2/CD85j, ILT4/CD85b and CD160 in Th1 cell clones (white bars), Th2 cell clones (grey bars) and Th17 cells (black bars). Results are expressed as MRFI. Mean of five different experiments ± SD is shown. **Panel C**. Blocking experiments were performed with CD4+ T cells stimulated with anti-CD3 mAb in the presence or absence of sHLA-G (100 nM) by adding anti-ILT2 or anti-ILT4 blocking mAbs, or isotype-matched control mAb. Percentage of CXCR3^+^ cells was evaluated by flow cytometry. Results are expressed as % of inhibition calculated as follows: [1−(% of positive cells with sHLA-G/% of positive cells w/o sHLA-G) ×100]. Histograms show mean and standard deviation of five different experiments performed. Statistical analysis was performed using t test. P value is shown where the difference is significant. **Panel D**. Representative histogram of FACS analysis of CXCR3 expression in CD4^+^ T cells stimulated with anti-CD3 mAb in the presence of anti-ILT2/CD85j agonist mAb (clone #F270) or isotype-matched control. A representative experiment out of three performed is shown. Dark profile indicated staining with specific mAb, whereas open profile indicated staining with isotype-matched mAb. Mean of MRFI values and standard deviations are indicated. Statistical analysis was performed using Mann-Whitney test. P values are indicated where the difference is significant.

The expression of three receptors for sHLA-G on the T cell surface was next investigated by flow cytometry. As shown in Fig. 5, panel A, all T cell fractions tested expressed CD160 (MRFI range 2,28–20,86). The expression of the inhibitory receptor ILT2/CD85j differed among the following T cell populations: i) high expression in CD8^+^ T cells and TCRγδ^+^ T cells (MRFI range 19,75–77,9), ii) intermediate expression in CD4^+^ T cells (MRFI range 7,11–12,4) and iii) low expression in T_FH_ cells (MRFI range 1,4–1,47). Expression of ILT4/CD85b was low in all T cell subsets (Fig. 4, panel A). The expression of all receptors was lower in polarized T-helper cells (CD160 MRFI range 1,31–1,63; ILT2 MRFI range 1,03–1,19; ILT4 range 1,08–1,16; Fig. 5 panel B). The expression of the sHLA-G receptors investigated was not modulated by sHLA-G treatment (data not shown).

To assess the role of ILT2/CD85j in the inhibition of T cell chemotaxis by sHLA-G, we performed five experiments in which CD4^+^ T cells were stimulated, and blocking antibodies against ILT2/CD85j or ILT4/CD85b, respectively, or isotypic control were added. CXCR3 expression was evaluated by flow cytometry as read-out of the experiment since this receptor was consistently downregulated by sHLA-G in different T cell populations.

As shown in Fig. 5, panel C, inhibition of CXCR3 expression on CD4^+^ T cells by sHLA-G was significantly reverted by adding a blocking mAb against ILT2/CD85j, as compared with isotypic control (% of inhibition 31,46 vs 47,55, p = 0,0052); conversely, no effect was observed in the presence of a blocking mAb against ILT4/CD85b.

These data indicated that sHLA-G inhibited T cell chemokine receptor expression by interacting mainly with ILT2/CD85j, although other sHLA-G receptor(s) are likely involved.

This conclusion was reinforced by additional experiments performed with an agonistic mAb F270 (kindly gifted by Dr.Daniela Pende) specific for ILT2/CD85j. CXCR3 expression on CD4^+^ T cells was dampened by treatment with anti-ILT2/CD85j agonistic mAb mimicking the results obtained upon sHLA-G treatment (mean MRFI±SD: isotype control 9.3±0.95; anti-ILT2/CD85j mAb 2.21±0.47; p = 0.022). Fig. 5, panel D shows a representative experiment out of three performed. Mean values and standard deviations are indicated.

### Pathways involved in sHLA-G driven intracellular signaling in T cells

The signaling pathways modulated by sHLA-G in stimulated CD4^+^ T cells was next investigated by Western-blot analysis of a panel of housekeeping and phosphorylated (p) proteins involved in HLA-G/ILT2 signaling[27]. These proteins have been chosen on the basis of data obtained by Durrbach *et al* (abstract at HLA-G International Conference, Paris 2009) and Naji *et al.* (J Exp Med, submitted) on B lymphocytes.

**Figure 6:**
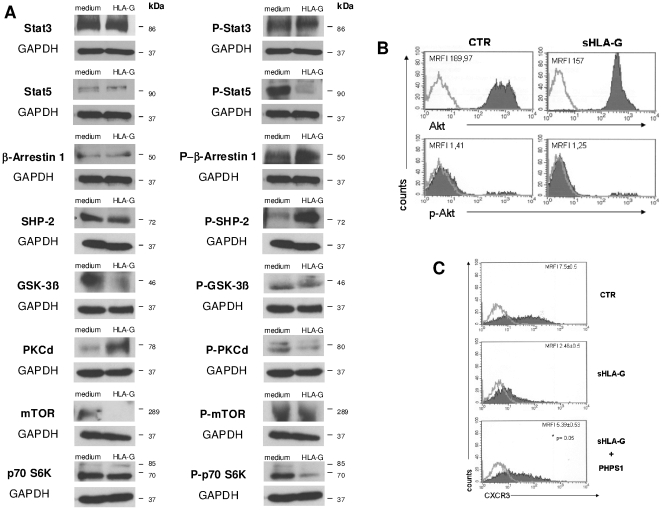
Western Blot analysis of sHLA-G intracellular signalling pathway. **Panel A.** Western blot analysis of total protein extracts from CD4+ T cells stimulated with anti-CD3 mAb in the presence or absence of sHLA-G (100 nM). Housekeeping proteins are shown in the left column, whereas phosphorylated protein are shown in the right column. GAPDH was evaluated as control. Molecular weight (KDa) for each band is indicated on the right. **Panel B.** Representative histogram of FACS analysis of Akt and p-Akt expression in CD4+ T cells stimulated with anti-CD3 mAb in the presence or absence of sHLA-G (100 ng/ml). A representative experiment out of three performed is shown. Dark profile indicated staining with specific mAb, whereas open profile indicated staining with isotype-matched mAb. MRFI values are indicated. **Panel C**. Representative histogram of FACS analysis of CXCR3 expression in CD4^+^ T cells stimulated with anti-CD3 mAb in the presence of medium alone, sHLA-G (100 ng/ml) and sHLA-G plus 10 µM PHPS1 (specific inhibitor of SHP-2 phosphatase). A representative experiment out of three performed is shown. Dark profile indicated staining with specific mAb, whereas open profile indicated staining with isotype-matched mAb. Mean of MRFI values and standard deviations are indicated. Statistical analysis was performed using Mann-Whitney test. P values are indicated where the difference is significant.

A representative experiment out of three performed is shown in Fig. 6, panel A. Quantification of proteins have been performed by densitometry autoradiography films. Results are summarized in Table 1.

**Table 1:** Quantification of proteins.

	ctr	SD	sHLA-G	SD	change	% FOLD
**p-STAT5**	**1,103**	*0,137*	**0,650**	*0,155*	**−0,453**	**−41,09**
**p-β-arrestin**	**0,860**	*0,136*	**1,053**	*0,054*	**0,193**	**+22,48**
**p-SHP-2**	**0,123**	*0,019*	**1,610**	*0,401*	**1,487**	**+1205,41**
**GSK-3-β**	**1,697**	*0,334*	**0,157**	*0,157*	**−1,540**	**−90,77**
**PKC-δ**	**0,433**	*0,095*	**1,160**	*0,404*	**0,727**	**+167,69**
**mTOR**	**1,513**	*0,621*	**0,000**	*0,000*	**−1,513**	**−100,00**
**p-p70 s6k**	**1,590**	*0,067*	**0,583**	*0,099*	**−1,007**	**−63,31**

Densitometry of autoradiography films was performed and relative density (RD) was calculated for control (ctr) and sHLA-G as follows: (density of specific band/density of GAPDH band). Change induced by sHLA-G was calculated as follows: (RD ctr-RD sHLA-G). % fold change induced by sHLA-G treatment was calculated as follows: [(RD sHLA-G - RD ctr)−1/RD ctr ×100]. RD, change and % fold change values are indicated only for proteins modulated by sHLA-G treatment. Mean values and standard deviation (SD) of three different experiments are shown.

p-Stat5 and p-p70 s6k, but not Stat5 and p70 s6k, respectively, were strongly downregulated by sHLA-G treatment. Up-regulation of p-SHP-2 and p-β-arrestin, but not of the corresponding housekeeping proteins, was detected in sHLA-G treated CD4^+^ T cells (Fig. 6, panel A). sHLA-G treatment decreased levels of p-PKC-δ and increased those of the corresponding housekeeping protein (Fig. 6, panel A).

Expression of GSK-3β and mTOR, but not that of their phosphorylated forms, was downregulated by sHLA-G. Expression of either p-Stat3 or Stat3 was unaffected by sHLA-G (Fig. 6, panel A).

Finally, no modulation of AKT (MRFI 189,97 vs 157) and p-AKT (MRFI 1,41 vs 1,25) expression by sHLA-G treatment was detected by flow cytometry. A representative experiment out of three performed is shown in Fig. 6, panel B.

Our data indicated that multiple intracellular signaling pathways in T lymphocytes can be affected by sHLA-G treatment.

We next analyzed whether hyper- phosphorylation of SHP-2 phosphatase was a crucial event in sHLA-G induced downregulation of chemokine receptors. Again, CXCR3 expression was analysed by flow cytometry as read-out of the experiments. Fig. 6, panel C, shows a representative experiment out of the three performed in which downregulation of CXCR3 expression in CD4^+^ T cells induced by sHLA-G was completely reverted when CD4^+^ T cells were pre-treated with PHPS1, a specific inhibitor of SHP-2[28] (mean MRFI±SD: CTR 17,5±0,5; HLA-G 2.46±0,5; HLA-G + PHPS1 5,39±0,53; p = 0,05).

## Discussion

In this paper, we describe a novel mechanism of immune modulation operated by sHLA-G molecules. This mechanism involves phenotypic downregulation of some chemokine receptors on different T cell populations associated with inhibition of chemotaxis of the latter cells to the respective ligands.

CD4^+^ T cells orchestrate adaptive immune responses by promoting B cell activation and differentiation to antibody secreting cells, maturation of cytotoxic T cells, activation of NK cells and macrophages. Activated CD4^+^ T cells are attracted by chemokine gradients to inflammatory sites where they migrate across endothelial vessels[29]. This latter process involves CD4^+^ T cell expression of different adhesion molecules, such as selectins and integrins, and different chemokine receptors[29]. Under polarizing antigenic stimulation, activated naïve CD4^+^ T cells differentiate into discrete subsets with distinctive patterns of cytokine production and effector functions[24]. These subsets display different chemotactic properties *in vitro* and *in vivo* related to the expression of distinctive sets of chemokine receptors[30].

In particular, i) Th1 T cells express CXCR3, CCR5 and CXCR6 and accumulate in inflammatory sites following gradients of chemokines such as CCL2 and CXCL10[31]; ii) Th2 T cells express CCR3, CCR4 and CCR8 and accumulate in inflammatory sites following gradients of chemokines such as CCL17 and CCL22[31] and iii) Th17 cells express CCR6 and CCR7 and are attracted by CCL20 and CCL19 or CCL21, respectively[32].

Here we show that CXCR3 expression was strongly downregulated by sHLA-G in total CD4+ T cells and Th1 cell clones, and accordingly chemotaxis of these cell fractions to the CXCR3 ligands CXCL10 and CXCL11 was significantly impaired by sHLA-G. In contrast, sHLA-G treatment did not affect CCR3, CCR4 or CCR8 expression by Th2 cell clones and CCR6 or CCR7 expression by Th17 cells.

Failure of sHLA-G to modulate Th2 cell associated chemokine receptors is consistent with the recent demonstration of high levels of sHLA-G in sera from allergic patients supporting the hypothesis that sHLA-G contributes to maintain Th2-polarized immune responses[33].

Th17 cells play a pivotal pathogenic role in mouse models of human rheumatoid arthritis[34], multiple sclerosis[35] and in patients with Crohn disease[36], in which CCR6 is crucial for Th17 cell attraction to sites of inflammation. Here we show for the first time that sHLA-G does not alter CCR6 expression in Th17 cells, suggesting that CCR6 driven chemotaxis is not amenable to inhibition mediated by sHLA-G.

We have here addressed also the effects of sHLA-G molecules on two populations of cytotoxic T cells, i.e. CTL and TCR Vδ2γ9 T cells, both of which control infections mediated by intracellular pathogens. In particular, CTL are specialized in the protection from viral infections[37], while TCR Vδ2γ9 T cells are mainly involved in protection from mycobacterial infections[38]. In analogy to that observed with CD4^+^ T cells and Th1 cell clones, both CTL and TCR Vδ2γ9 T cells showed strong downregulation of CXCR3 by sHLA-G associated with inhibition of chemotaxis to CXCL10 and CXCL11. The finding that CXCR4 expression was up-regulated by sHLA-G in TCR Vδ2γ9 T cells without an increase of the migration of the latter cells towards CXCL12 was not surprising, since several examples in the literature have shown that modulation of chemokine receptor expression is not always coupled with changes in receptor function[39].

sHLA-G is detected in sera from normal donors and is physiologically produced by myeloid cells such as monocytes and dendritic cells, as well as by activated endothelial cells[25]. Furthermore, serum sHLA-G concentration is modulated in different pathological conditions. In particular, serum sHLA-G levels increase in patients with solid and haematological tumors, allergy and viral infections, and decrease in patients with auto-immune disorders[25]. Moreover, the concentration of sHLA-G tested in our experiments *in vitro* (100 ng/ml) was observed in sera from patients with solid and haematological malignancies, whereas concentration of sHLA-G in sera healthy subjects is in general below 20 ng/ml[40], [41]. Therefore, our results may have pathophysiological relevance since disease-related fluctuations in sHLA-G serum levels may translate into modulation of T cell migration to inflamed tissues. In this connection, we have demonstrated that CXCR3 is the main target of sHLA-G mediated inhibition in different T cell populations. CXCR3, together with CCR5, controls Th1 cell migration[42] and is therefore involved in a broad spectrum of functional activities of these cells. Th1 cells collaborate with TCR Vδ2γ9 T cells in the control of mycobacterial infections[43]. Therefore, it is tempting to speculate that sHLA-G, by dampening CXCR3 mediated chemotaxis, modulates the recruitment of these cell populations to granulomatous mycobacterial lesions, perhaps limiting excessive tissue damage. Likewise, in the course of viral infections, attraction of CTL to infected tissues by CXCR3 ligands may be modulated by sHLA-G whose serum levels are increased in patients with different types of viral infection.

Another implication of our results deals with anti-tumor immune responses in which both CTL and TCR γδ T cells play important roles. Attraction of these cells to the tumor mass is operated in part by CXCR3 ligands expressed in variable amounts in the tumor microenvironment by tumor cells or stromal cells[44]. Since sHLA-G levels are strongly increased in many malignancies, dampened recruitment of CTL and TCR γδ T cells to the tumor site is likely to take place *in vivo* and provide a novel mechanism of sHLA-G related immunosuppression.

Several examples in literature support our data. It has been reported that CXCR3 is downregulated in several pathological conditions, in which serum sHLA-G levels are increased, such as allergy[45], T cell lymphoma[46]and multiple sclerosis [47]. Conversely, CXCR3 has been found to be upregulated in diseased associated with decerased levels of serum sHLA-G, such as rheumatoid arthritis and lupus [48].

Here we show for the first time that CXCR5 expression and CXCL13 driven chemotaxis were strongly downregulated by sHLA-G in T_FH_ cells, a subset of CD4^+^ cells coexpressing CXCR5 and ICOS that circulate from peripheral blood to secondary lymphoid organs, that may be developmentally related to Th1, Th2 or Th17 cells[49]. T_FH_ cells exert a potent helper function for centrocyte differentiation to plasma cells in the light zone of the GC, where we detected expression of sHLA-G molecules that was also observed in the subepithelial area of tonsil.

Taken together, these findings support the conclusion that migration of T_FH_ cells in the light zone of the germinal centre may be modulated by a gradient of sHLA-G and this can in turn interfere in T_FH_-dependent plasma cell differentiation. Based upon recent studies of dynamic imaging of GC cell trafficking in mice, it has been proposed that positive selection of GC B cells depends not only on recognition of antigen presented by follicular dendritic cells, but also on competition for help provided by T_FH_ cells[50]. Thus, in principle, modulation of CXCR5 expression and function by sHLA-G may impact indirectly on GC B cell positive selection.

sHLA-G mediated inhibition of T cell chemokine receptor expression and chemotaxis was found to depend mainly on the inhibitory receptor ILT2/CD85j. The signalling pathway initiated by sHLA-G interaction with ILT2/CD85j involved modulation of phosphorylation of SHP-2, Stat5 and p70 s6k. sHLA-G induced over-phosphorylation of SHP-2 together with reduced phosphorylation of Stat5. SHP-2 is a tyrosine phosphatase that upon phosphorylation by ligand-stimulated inhibitory receptors such as ILT2/CD85j and ILT4/CD85b can de-phosphorylate and inactivate Stat5, leading to reduced transcription of several[51] genes including some implicated in cell motility[52]. Indeed, we demonstrated that SHP-2 plays a pivotal role in sHLA-G induced downregulation of T cell CXCR3 using the specific SHP-2 inhibitor PHPS1[28].

Numerous signalling pathways converge on p70 s6k that, upon phosphorylation, participate in the control of cell cycle progression[53]. We also demonstrated that sHLA-G induced over-phosphorylation of β-arrestin, that in its phosphorylated form binds chemokine receptors and promotes their internalization, thus preventing further interactions with their ligands[54]. Surprisingly, sHLA-G modulated expression of the housekeeping but not the phosphorylated mTOR, GSK-3β and PKC-δ proteins. The functional significance of these findings is unknown.

**Figure 7:**
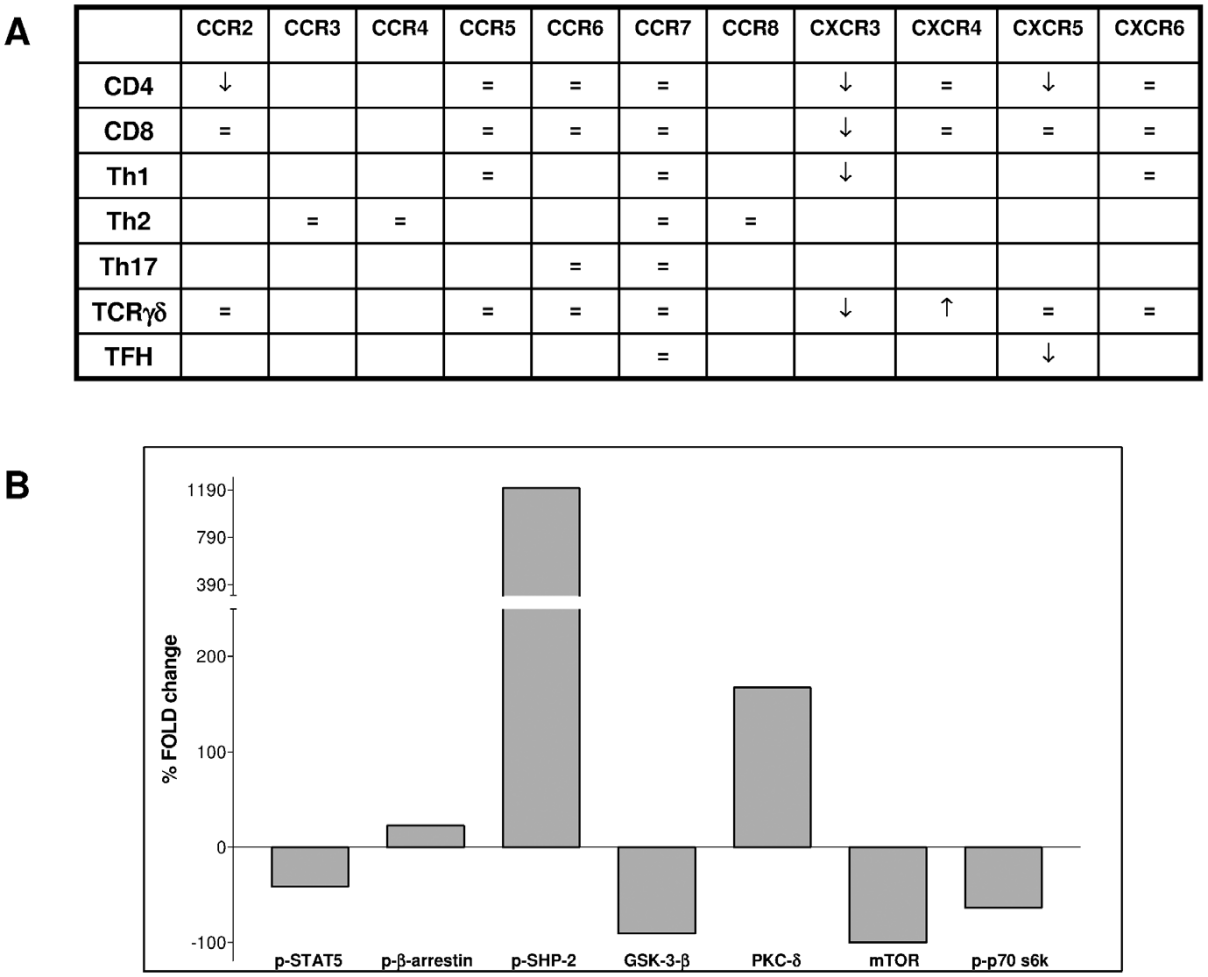
Summary of data. **Panel A**. Table summarized data obtained on the modulation of chemokine receptors expression in different T cell populations. Upward arrows indicated upmodulation of the receptors. Downward arrows indicated downmodulation of the receptors. **Panel B**. Histogram summarized data obtained in Western Blot analysis on CD4 T cells stimuated in the presence or absence of sHLA-G. Densitometry of autoradiography films was performed and relative density (RD) was calculated for control (ctr) and sHLA-G as follows: (density of specific band/density of GAPDH band). Change induced by sHLA-G was calculated as follows: (RD ctr-RD sHLA-G). % fold change induced by sHLA-G treatment was calculated as follows: [(RD sHLA-G - RD ctr)-1/RD ctr ×100].

The main findings of this study have been summarized in Figure 7.

In conclusion, we have shown that sHLA-G performs a novel immunomodulatory effect on T cells, downregulating the expression and function of chemokine receptors. Such inhibition can affect the recirculation of T lymphocytes between peripheral blood, secondary lymphoid organs and inflamed tissues, leading to important repercussions on their effector functions.

## Materials and Methods

### HLA-G1/G5 production

Recombinant HLA-G1/G5 protein was produced in the human lymphoblastoid cell line 721.221.G1 (kindly provided by Dr. Francesco Puppo, DIMI, Genoa) by transfection of the 721.221 parental cell line with human HLA-G1 cDNA[55].

Supernatants were collected from the 721.221.G1 cell line after 72 h culture in RPMI 10% FBS at 37°C and 5% CO_2_, and subsequently purified using MEM-G/9 monoclonal antibody and goat anti-mouse beads (Immunotech, Praha, Czech Republic). Soluble HLA-G was quantified by HLA-G1/G5 specific ELISA.

sHLA-G ELISA was performed using MaxiSorp Nunc-Immuno 96 microwell plates (Nunc A/S, Roskilde, Denmark) coated overnight at 4°C with mAb MEM-G/9 (Exbio Praha, Czech Republic; 10 µg/ml) in 0.001 M PBS, pH 7.4 (EuroClone SpA, PV, Italy). After three washes with PBS 0.05% Tween 20 (washing buffer), plates were saturated with 200 µl/w of PBS 2% BSA (Sigma, St.Louis, MO, USA) for 30 min at RT.

100 µl of test samples or standard (serial dilutions of calibrated 721.221.G1 cell line supernatant) were added to each well and incubated at RT for 1 h. Plates were washed three times with washing buffer, and then incubated with 100 µl/well of biotinylated anti-β2m mAb NAMB-1 (1 µg/ml) at RT for 1 h (kindly donated by Dr. Soldano Ferrone). After three washes, plates were incubated at RT for 1 h with streptavidin-horse radish peroxidase (GE Healthcare, Chalfont St. Giles, United Kingdom) 1∶4000 in PBS 0.1% Tween 20, 0.1% BSA, for 1 h at RT. After three additional washes, plates were incubated with 3′-3′-5′-5′ Tetramethylbenzidine (TMB, Sigma) for 5 min at RT. H_2_SO_4_ 5 M (100 µl/w) was then added, and optical densities were measured at 450 nm.

The assay's lowest threshold was 1,95 ng/ml of sHLA-G. Each sample was tested in duplicate.

### Cell isolation and culture

The study was approved by the Ethical Committee of the G. Gaslini Institute, Genoa, Italy. Surgically removed tonsils and normal peripheral blood (PB) samples were obtained following written informed consent. Mononuclear cells (MNC) were isolated by Ficoll-Hypaque (Sigma) density gradient.

CD4^+^ and CD8^+^ T cells were isolated from PB samples using anti-CD4 or anti-CD8 microbeads (Myltenyi Biotec, Bergisch Gladbach, Germany) following manufacturer's protocol.

T cell clones were generated by cloning at limiting dilution as previously described[56].

Th1 T cell clones were obtained from normal donors' PBMNC after stimuation with *Candida Albicans* bodies for 6 days as reported[57]. Th2 T cell clones were obtained from PBMNC of donors with allergic sensistization to *Dermatophagoides Pteronissinus* (DP) after stimulation with DP extract (Stallergenes, France) 20 µg/ml for 6 days as reported[57]. After stimulation, T cell blasts were separated using PERCOLL (GE Healthcare) density gradient, and were then expanded in RPMI 10% FBS +200 U/ml IL-2 (Chiron, Milano, Italy). Th17 T cells were obtained from normal donors' PBMNC using Th17 expansion kit (Myltenyi Biotec) following manufacturer's protocol.

T_FH_ cells were isolated from human tonsils mononuclear cells by immunomagnetic selection of ICOS^+^ T cells, using anti-ICOS mAb (Santa Cruz Biotechnology, CA, USA) and anti-mouse IgG1 microbeads (Myltenyi Biotec). These fractions contained >95% of CD4^+^/ICOS^+^/CXCR5^+^ T_FH_ cells.

TCR TCRγδ T cells circulate in peripheral blood (3–5% of PBMNC) and express a peculiar TCR composed of γ and δ chains, that recognize phosphoantigens. Most circulating TCRγδ T cells show the Vδ2γ9 rearrangement[17]. TCR Vδ2γ9 T cells were obtained by stimulating PBMNC from normal donors with 5 uM n-bisphosphonate zoledronate (Novartis) for 7 days, as described[58]. After stimulation, 92% of these cells were TCR Vδ2γ9 ^+^ T cells.

All these T cell populations were stimulated *in vitro* for 48 h in RPMI 10% FBS at 37°C and 5% CO_2_ with anti-CD3 mAb (OKT3, coated O.N. on 96 well plates) in the presence or absence of sHLA-G (100 ng/ml) before being subjected to flow cytometric analysis or *in vitro* migration assay.

### Antibodies and flow cytometry

The following mAbs were used: anti-CXCR4 PE (clone #12G5), anti-CXCR5 PE (clone #51505), anti-CCR7 APC (clone #150503), anti-CCR6 PE (clone #53103), anti-CCR2 PE (clone #48607), anti-CXCR3 FITC (clone #49801), anti-CXCR6 PE (clone #56811), anti-CCR5 PE (clone #45531) (R&D System Inc., Minneapolis, MN, USA), anti-ICOS (clone # ANCC6C6-A3) Santa Cruz Biotechnology), anti-CD4 FITC (clone# RPA-T4) and anti-TCR γδ PE (clone# GL3) (Becton Dickinson, NJ, USA).

Cells were stained with fluorochrome-conjugated mAbs or with isotype and fluorochrome-matched control antibodies, and were run on a FACSCalibur (Becton Dickinson). 10^4^ events were acquired and analyzed using the CellQuest software (Becton Dickinson).

### Blocking experiments

Blocking experiments were performed by stimulating cells as described above, adding 1 µg/10^6^ cells of blocking antibodies anti-ILT2/CD85j (clone #292319) or anti-ILT4/CD85b (clone #287219, R&D system) or isotype-matched control (Invitrogen, CA, USA).

The specific SHP-2 phosphatase inhibitor PHPS1[28] was purchased from Sigma Aldrich. Cells were cultured for 2 h at 37°C in the presence or absence of PHPS1 (10 µM), and then stimulated as described above. Agonistic mAb (clone # F278) [59] specific for ILT2/CD85j was kindly gifted by Dr. Daniela Pende (IST, Genoa, Italy). Cells were stimulated with anti-CD3 mAb or PHA for 72 h, in the presence or absence of 5 µl/10^6^ cells of anti-ILT2/CD85j monoclonal antibody.

### Chemotaxis

Chemotaxis was investigated using 5 µm pore-size transwell plates (Costar, Cambridge, MA) as reported[60]. Five ×10^5^ cells were dispensed in the upper chamber, whereas chemokines or medium alone were added to the lower chamber. CCL2, CCL8, CCL21, CXCL10, CXCL11 (Immunotools, Friesoythe; Germany) CXCL12 and CXCL13 (Abnova, Heidelberg, Germany) were tested at 300 ng/mL[60] following preliminary titration experiments. Plates were incubated 2 h at 37°C. Migrated cells were collected and counted, and migration index was calculated as follows: (n° of migrated cells/n° of dispensed cells*100). Migration index obtained with medium alone was subtracted from each value.

### Immunohistochemical staining of human tonsil

Immunohistochemical staining of tissue sections was performed using the Envision System HRP mouse (Dako, Glostrup, Denmark). Briefly, 5 µm thick sections were cut from formalin fixed, paraffin embedded blocks, deparaffinized with xylene and rehydrated by passages through decreasing concentrations of ethanol (from 100% to 80%). Endogenous peroxidase activity was blocked by a 30 min incubation at room temperature with methanol containing 3% H_2_O_2_. Tissue sections were then incubated at 98°C for 40 min in citrate buffer (pH 6.0) for antigen retrieval (ChemMate, Dako). After rinsing in Optimax™ Wash Buffer (Menarini Diagnostics, Firenze, Italy), tissue sections were incubated 1 hour at room temperature with optimal amounts of anti-HLA-G (1∶25 MEM-G2, Exbio) or isotype control (mouse IgG1, Invitrogen). Tissue sections were washed twice in Optimax™ Wash Buffer and incubated for 30 min at room temperature with Dako Envision System horse radish peroxidase (HRP) Mouse. After washing in Optimax™ Wash Buffer, peroxidase activity was detected by incubating tissue sections for 6–10 min at room temperature with Dako Liquid DAB Substrate Chromogen System (Dako). Tissue sections were counterstained with Mayer's hematoxylin (Sigma).

### Western blot

Western blot analysis was performed on total extracts from 30*10^6^ of CD4+ T cells stimulated as described above in presence or absence of sHLA-G (100 ng/ml). Protein extracts were obtained using Cell Extraction Buffer buffer (BioSource International, CA, USA) plus protease inhibitor cocktail (Sigma). Protein quantification was performed by BCA assay (Sigma).

Total cell lysates were prepared and analyzed by western blot analysis as described earlier[61]. Briefly, cells were lysed with Cell Extraction Buffer (BioSource International) plus protease inhibitor cocktail (Sigma). Protein lysates (70 ug per lane) were resolved on SDS 12,5% polyacrylamide gels and were transferred to nitrocellulose membranes. The membranes were then incubated with the following mouse monoclonal antibodies: anti-Stat3, anti-phospho Stat3, anti-Stat5, anti-phospho Stat5, anti β-arrestin1, anti-phospho β-arrestin1, anti-SHP-2, anti phospho SHP-2, anti-GSK3β, anti-phospo GSK-3β, anti PKCδ, anti-phospo PKCδ, anti mTOR, anti-phospo mTOR, anti-p70 S6K, anti-phospo p70 S6K (Cell Signaling, MA, USA).

Peroxidase-conjugated goat anti-mouse and anti-rabbit polyclonal antisera were used as secondary reagents (Upstate/Millipore, MA, USA and Santa Cruz Biotechnology, respectively).

Immune complexes were visualized with the use of a Supersignal West Pico Chemiluminescent Substrate (Pierce, Rockford, USA) according to the manufacturer's instructions, and were normalized to internal controls (a rabbit monoclonal antibody against glyceraldehyde-3-phosphate dehydrogenase (Cell signaling). Protein levels were quantified by scanning densitometry of the autoradiography films using VersaDoc 3000 Gel Imaging System (BioRad, CA, USA) and normalized over (ratio) the housekeeping protein levels.
